# Real-world experience of IL-17Ai drug survival in a large cohort of axial spondyloarthritis and psoriatic arthritis

**DOI:** 10.1093/rap/rkae018

**Published:** 2024-02-15

**Authors:** Jake Weddell, Naw Ra Aung Din, Stephanie R Harrison, Xabier Michelena, Dennis McGonagle, Andrew Barr, Claire Vandevelde, Jane Freeston, Helena Marzo-Ortega

**Affiliations:** NIHR Leeds Biomedical Research Centre, Leeds Teaching Hospitals Trust, Leeds, UK; Leeds Institute of Rheumatic and Musculoskeletal Medicine, University of Leeds, Leeds, UK; Department of Rheumatology, Leeds Teaching Hospitals NHS Trust, Chapel Allerton Hospital, Leeds, UK; Department of Rheumatology, Leeds Teaching Hospitals NHS Trust, Chapel Allerton Hospital, Leeds, UK; NIHR Leeds Biomedical Research Centre, Leeds Teaching Hospitals Trust, Leeds, UK; Leeds Institute of Rheumatic and Musculoskeletal Medicine, University of Leeds, Leeds, UK; Department of Rheumatology, Leeds Teaching Hospitals NHS Trust, Chapel Allerton Hospital, Leeds, UK; Leeds Institute of Rheumatic and Musculoskeletal Medicine, University of Leeds, Leeds, UK; Rheumatology Unit, Vall d’Hebron Hospital Universitari, Vall d’Hebron Barcelona Hospital Campus, Barcelona, Spain; NIHR Leeds Biomedical Research Centre, Leeds Teaching Hospitals Trust, Leeds, UK; Leeds Institute of Rheumatic and Musculoskeletal Medicine, University of Leeds, Leeds, UK; Department of Rheumatology, Leeds Teaching Hospitals NHS Trust, Chapel Allerton Hospital, Leeds, UK; Leeds Institute of Rheumatic and Musculoskeletal Medicine, University of Leeds, Leeds, UK; Department of Rheumatology, Leeds Teaching Hospitals NHS Trust, Chapel Allerton Hospital, Leeds, UK; Leeds Institute of Rheumatic and Musculoskeletal Medicine, University of Leeds, Leeds, UK; Department of Rheumatology, Leeds Teaching Hospitals NHS Trust, Chapel Allerton Hospital, Leeds, UK; NIHR Leeds Biomedical Research Centre, Leeds Teaching Hospitals Trust, Leeds, UK; Leeds Institute of Rheumatic and Musculoskeletal Medicine, University of Leeds, Leeds, UK; Department of Rheumatology, Leeds Teaching Hospitals NHS Trust, Chapel Allerton Hospital, Leeds, UK; NIHR Leeds Biomedical Research Centre, Leeds Teaching Hospitals Trust, Leeds, UK; Leeds Institute of Rheumatic and Musculoskeletal Medicine, University of Leeds, Leeds, UK; Department of Rheumatology, Leeds Teaching Hospitals NHS Trust, Chapel Allerton Hospital, Leeds, UK

**Keywords:** IL-17Ai, drug survival, psoriatic arthritis, axial spondyloarthritis, real-world evidence

## Abstract

**Objective:**

The aim was to assess the use and drug survival of IL-17Ai in a real-world cohort of axial SpA (axSpA) and PsA patients.

**Methods:**

Patients ever commenced on an IL-17Ai (secukinumab or ixekizumab) for axSpA or PsA at the Leeds Specialist Spondyloarthritis Service were identified. Demographics, IL-17Ai treatment length and reason for cessation were collected. Drug survival data were plotted as a Kaplan–Meier curve, with log rank test of median survival compared between axSpA and PsA. Cox regression analysis was performed to investigate the relationship between diagnosis and length of drug survival.

**Results:**

In total, 228 patients (91 axSpA and 137 PsA) were exposed to IL-17Ai. Drug survival for all patients at 12 months was 69% (95% Confidence Interval (CI) 63, 75%) and at 24 months 60% (95% CI 54, 67%). In axSpA and PsA, drug survival at 12 months was 63% (CI 54, 74%) and 73% (CI 66, 81%), respectively, and at 24 months it was 53% (CI 44, 65%) and 65% (CI 57, 75%), respectively. Median survival did not differ significantly between both diseases (log rank test 0.65). There was no association between diagnosis and survival (hazard ratio 0.92, 95% CI 0.63, 1.33), including when adjusting for age, previous biologic DMARD usage and sex (hazard ratio 0.89, 95% CI 0.61, 1.13).

**Conclusion:**

This is the first study, to our knowledge, to analyse and compare real-world IL-17Ai drug survival in patients with axSpA and PsA from a single centre. We demonstrate that there is no difference in IL-17Ai survival rates and no relationship between diagnosis and drug survival. These results contribute to the body of real-world evidence confirming the role of IL-17Ai in the management of axSpA and PsA.

Key messages

- IL-17Ai drug survival is not dependent on diagnosis in SpA.
- IL-17Ais are well tolerated in patients with axSpA and PsA.
- This study adds to the real-world evidence supporting IL-17Ai usage in SpA.

## Introduction

The SpAs are a heterogeneous group of immune-mediated diseases characterized by inflammation of the peripheral and/or axial joints, enthesis and surrounding soft tissues (dactylitis) and extra-musculoskeletal manifestations, including skin/nail psoriasis, IBD and uveitis [1]. The commonest subtypes are axial SpA (axSpA) [2], which can be sub-classified according to the presence or absence of radiographic sacroiliitis [radiographic (r-)axSpA] and non-radiographic (nr-)axSpA, and PsA, which can have a peripheral and axial phenotype [3].

The IL-17/23 axis is widely implicated in the pathophysiology of SpA. IL-17A is produced by activated Th17 cells present in the joints and the skin, and it stimulates immune cells to produce multiple pro-inflammatory mediators [4]. The IL-17A inhibitors secukinumab and ixekizumab have demonstrated safety and efficacy in randomized controlled trials for r-axSpA, nr-axSpA and PsA, leading to their approval by NICE in the UK in 2016 [5] and 2018, respectively [6].

Yet, there are limited real-world data on drug survival, particularly across the whole of the SpA spectrum. Current studies use relatively small cohorts of patients, analyse both drugs (secukinumab and ixekizumab) separately, have limited follow-up and do not necessarily compare drug survival across the whole spectrum of both axSpA and PsA [7–13]. Here, we report our experience on long-term outcomes and drug survival of patients with axSpA and PsA treated with secukinumab and ixekizumab from a single centre in the UK.

## Methods

We undertook an audit of SpA patients attending the Leeds Teaching Hospitals Trust Specialist Spondyloarthritis Service, who were treated with either secukinumab or ixekizumab up to December 2022. No formal ethical approval was required for this service evaluation exercise, and the project was registered with the information governance team to ensure compliance with local processes. All patients listed in our in-house biologic prescription registry were included. We conducted a retrospective review of the medical records to summarize the relevant baseline and clinical data, including patient demographics (age, sex, BMI, smoking status and co-morbidities) and relevant clinical characteristics, including diagnosis (axSpA *vs* PsA) and disease duration, in addition to treatment data, including previous exposure to biologic DMARDs (bDMARDs), the duration of IL-17Ai and the reasons for discontinuation, where applicable.

Reasons for treatment cessation were broadly defined as attributable to loss of efficacy, patient-reported adverse effects and other causes. Owing to outcome measures being unavailable in many patients, loss of efficacy was determined by the opinion of the treating rheumatologist. Loss of clinical efficacy was then divided further into primary non-response, when treatment cessation was attributable to inefficacy or loss of efficacy within 6 months of drug initiation, and secondary non-response, when treatment cessation was attributable to loss of efficacy after 6 months.

All data were analysed using R Studio (2023.06.0 + 421 ‘Mountain Hydrangea’ Release for macOS). Data are reported as the median and interquartile range (IQR) for continuous variables and percentage/proportions for categorical variables. Drug survival was plotted using Kaplan–Meier curves, and time to loss of response was analysed using Cox proportional hazard ratios, adjusting for age, previous dDMARD exposure and sex.

## Results

Overall, 228 patients were exposed to IL-17Ai therapy. Of these, 91 had a diagnosis of axSpA and 137 of PsA. Patients with axSpA were more likely to be male (axSpA 59% *vs* PsA 43%), to be HLA-B27-positive (axSpA 75% *vs* PsA 12%), to have symptom onset at a younger age (mean onset: axSpA 32 years *vs* PsA 39 years), to be bDMARD-naïve (axSpA 29% *vs* PsA 22%), to be a current or previous smoker (axSpA 59% *vs* PsA 34%) and to have fewer extra-musculoskeletal manifestations (Table 1). Both groups had similar BMI (axSpA 28 kg/m^2^*vs* PsA 30 kg/m^2^) and had failed similar numbers of bDMARDS [median (IQR): axSpA 2 (1–3) *vs* PsA 2 (1–3)] before exposure to IL-17Ai.

**Table 1.** Demographics and disease characteristics

Characteristic	Overall	PsA	axSpA
	(*n* = 228)	(*n* = 137)	(*n* = 91)
Age, median (IQR), years	47 (37–57)	47 (36–60)	48 (36–55)
Male [*n* (%)]	114 (50.0)	60 (43.8)	54 (59.3)
Current/ex-smoker [*n* (%)]	101 (44.3)	47 (34.3)	54 (59.3)
BMI, median (IQR), kg/m^2^	29 (25–34)	29 (26–34)	28 (24–32)
Disease duration, median (IQR), years	4 (3.1–4.9)	4 (3.3–5.0)	4 (3.0–4.7)
HLA-B27 positive [*n* (%)]	86 (37.7)	17 (12.4)	69 (75.8)
Psoriasis [*n* (%)]	148 (64.9)	130 (94.9)	18 (19.8)
IBD [*n* (%)]	4 (1.8)	1 (0.1)	3 (3.3)
Uveitis [*n* (%)]	35 (15.4)	15 (10.9)	20 (22.0)
Raised CRP [*n* (%)]	150 (65.8)	86 (62.8)	64 (70.3)
Secukinumab [*n* (%)]	159 (69.7)	81 (59.1)	78 (85.7)
Ixekizumab [*n* (%)]	69 (30.2)	56 (40.1)	13 (14.3)
Biologic exposed [*n* (%)]	172 (75.4)	107 (78.1)	65 (71.4)
Previous biologics, median (IQR), *n*	2 (1–3)	2 (1–3)	2 (1–3)
r-axSpA [*n* (%)]	NA	NA	64 (70.3)
nr-axSpA [*n* (%)]	NA	NA	27 (29.6)
**Reasons for drug discontinuation (IL-17i)**	**(*n* = 112)**	**(*n* = 63)**	**(*n* = 49)**
Primary non-response [*n* (%)]	31 (27.7)	12 (19.0)	19 (38.8)
Secondary non-response [*n* (%)]	41 (36.6)	27 (42.9)	14 (28.5)
Side effects [*n* (%)]	26 (23.2)	15 (23.8)	11 (22.4)
Other [*n* (%)]	14 (12.5)	9 (14.3)	5 (10.2)

axSpA: axial SpA; IQR: interquartile range; NA: not applicable; nr-axSpA: non-radiographic axSpA; r-axSpA: radiographic axSpA.

In terms of IL-17Ai usage, secukinumab was the most frequently used (*n* = 159) *vs n* = 69 on ixekizumab. Secukinumab usage was similar between axSpA and PsA patients (78 axSpA *vs* 81 PsA); however, ixekizumab was much more frequently used in PsA patients than in axSpA (13 axSpA *vs* 56 PsA).

**Figure 1.**
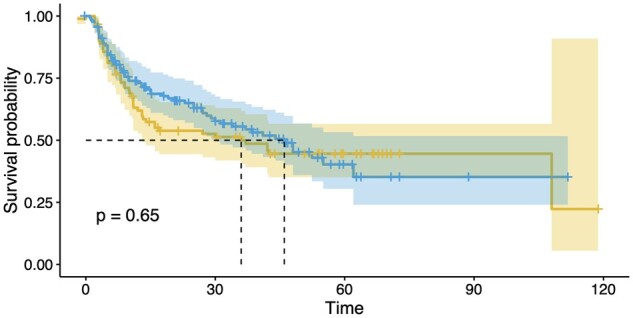
Kaplan–Meier curve showing median survival of IL-17Ais in radiographic axial SpA/AS (yellow) and PsA (blue) There were no statistically significant differences in median drug survival at 1 year, even with adjustment for age, previous biologic DMARD use and sex

Drug survival for all patients at 12 months was 69% (95% CI 63, 75%) and at 24 months 60% (95% CI 54, 67%). When compared according to diagnosis, 12 months drug survival or survival in axSpA *vs* PsA were 63% (95% CI 54, 74%) and 73% (95% CI 66, 81%), respectively, and at 24 months it was 53% (95% CI 44, 65%) and 65% (95% CI 57, 75%), respectively (Fig. 1). There was no significant difference in median drug survival between groups (log rank test, *P* = 0.65). Furthermore, there was no significant association between diagnosis and drug survival (hazard ratio 0.92, 95% CI 0.63, 1.33), including when adjusting for age, previous bDMARDs and sex (hazard ratio 0.89, 95% CI 0.61, 1.13). Finally, there was no difference in drug survival in bDMARD-naïve *vs* exposed groups when analysing the PsA and axSpA subgroups separately (log rank test *P* = 0.38 and *P* = 0.57, respectively).

Clinical non-response was the most common reason for treatment discontinuation (Table 1). Primary non-response was higher in axSpA than PsA (axSpA 39% *vs* PsA 19%) and, conversely, secondary non-response was higher in PsA (axSpA 29% *vs* PsA 43%). Drug discontinuation owing to adverse events was seen at similar rates with both drugs in each disease (axSpA 22% *vs* PsA 24%). The commonest reasons for discontinuation were infection [23%, 6 of 26; of which 8% (2 of 26) were persistent superficial fungal infections] and injection site reactions [15% (4 of 26)]. Two patients developed colitis; one biopsy-proven Crohn’s disease-related IL-17i use, the other biopsy indeterminant (perhaps infective, perhaps IBD). Less common side effects (observed in fewer than four cases) were: alopecia universalis, blepharitis, burning skin sensation, fatigue, gastrointestinal (nausea, vomiting, diarrhoea), headaches, mouth ulcers, night sweats, psoriasis flare, rash and visual disturbances.

## Discussion

We present data on real-world IL-17Ai drug survival in SpA and assess the impact of diagnosis on drug survival. We demonstrate that IL-17Ais have high levels of drug survival and that this is not affected by the underlying diagnosis, including when corrected for differences in age, sex and previous bDMARD use, which is of relevance when treating a bDMARD-experienced population. The commonest reason for treatment cessation was lack of clinical efficacy, with adverse effects infrequently being the primary cause.

To our knowledge, this study is the first to analyse drug survival of currently available IL-17Ais across both indications in SpA; however, many real-world studies have presented data on secukinumab alone across each indication. Alonso *et al.* [9] reported data on secukinumab survival in axSpA and PsA from 154 patients in northern Spain, showing overall similar 1-year survival to our study (66% *vs* 69%), but significantly lower 2-year survival (43% *vs* 60%), despite having more biologic-naïve participants. The FORSYA multicentre study in France identified 904 patients with axSpA [10] and 475 with PsA [13] who were treated with secukinumab and found a 59% 1-year survival rate for axSpA and 63% 1-year survival for PsA patients. Data from the CANSPA network [8] found a 12-month 74% survival for 213 PsA patients and a 63% survival for 146 axSpA patients. Baseline demographics varied significantly within each of these studies, and larger registry-based studies are needed to identify predictors of drug survival and overall real-world IL-17Ai survival.

Comparison of drug survival between secukinumab and ixekizumab was, unfortunately, not possible in our report owing to the low usage of ixekizumab in patients with axSpA. Ixekizumab was first licensed for use in axSpA in the UK in 2021, compared with 2016 for secukinumab, which most probably explains its less frequent usage for this condition. Data on real-world ixekizumab survival are sparse, with no studies exploring survival in axSpA. In PsA, the data are limited and vary significantly. The Spanish multicentre pro-Stip [11] study of 89 patients with PsA found ixekizumab survival rates of 85% at 48 weeks and 69% at 104 weeks, whereas a single-centre [12] study of 72 patients found 65% survival at 1 year and 57% at 2 years. Given that ixekizumab continues to be used in clinical practice for the treatment of PsA and axSpA, further evidence will develop, and a comparison between IL-17Ais might become possible.

Interestingly, the survival of IL-17Ai in patients with SpA is significantly lower than the survival reported in the psoriasis population. In a study of the BADBIR UK psoriasis biologic database [14], secukinumab had a 1-year survival function of 0.85 and a 2-year survival function of 0.75 in patients with moderate-to-severe psoriasis, with the presence of PsA being a negative predictor for drug survival. Data from the DANBIO and DERMBIO [15] Danish biologic registries showed that median drug survival for secukinumab was greater in psoriasis than in PsA and axSpA in bio-naïve and bio-exposed patients. Most recently, the SERENA study in the UK reported secukinumab survival rates in PsA/r-axSpA of 91.0% and 89.2% at 12 months, respectively; however, unlike in our cohort, patients were only entered into SERENA after successfully tolerating 4 months of therapy, and thus any patients with true primary non-response were excluded [16]. There are many factors that might contribute to worse survival rates in SpA compared with psoriasis, including the effects of diagnostic delay, co-existing joint disease and non-inflammatory pain pathways. A recent Swedish registry study [17] showed the presence of extra-musculoskeletal manifestations, particularly uveitis and psoriasis, in axSpA negatively impacted upon TNF inhibitor survival. A significant number of patients in our study had extra-musculoskeletal manifestations of SpA, and the poorer survival compared with the psoriasis population might reflect the complexities of treating multiple tissue manifestations of the SpA spectrum with a single agent.

Loss of clinical efficacy was the commonest reason for stopping IL-17Ai in our cohort. Although 1-year, 2-year and median survival showed no significant difference between axSpA and PsA, there were differences in primary and secondary non-response rates. Patients with axSpA predominantly experienced primary non-response, whereas patients with PsA were more likely to experience secondary non-response. This might reflect the positive impact of IL-17Ais upon psoriasis, which was present in the majority of our PsA patients, leading to longer initial survival. IL-17Ais were well tolerated in our study, with only 23% of patients ceasing treatment owing to side effects or adverse events, which is lower than that reported in other cohorts [9, 11]. No difference was seen between the bDMARD-naïve and or previously exposed population or according to the number of bDMARD agents previously used.

Our study has some limitations. Patients were identified only if they received IL-17Ai through the rheumatology prescribing service; therefore, those who were prescribed through dermatological or other services were not included. Data on IL-17Ai dose, treatment intervals and concomitant medications were not available, and therefore we were unable to assess the impact of different treatment regimens or dosing regimens upon survival. Finally, clinical outcome measures, including Psoriasis Area and Severity Index (PASI), BASDAI and swollen/tender joint counts were not widely available, reflecting a common scenario in clinical practice, which limits any assessment of efficacy or whether treatment cessation was linked to failure in a particular SpA manifestation.

## Conclusion

This is the first study, to our knowledge, to analyse and compare real-world IL-17Ai drug survival in patients with axSpA and PsA from a single centre. We demonstrate that there is no difference in IL-17Ai survival rates between both conditions and no relationship between diagnosis and drug survival, even when adjusting for differences in baseline and disease characteristics. The lower survival figures at 2 years might point towards the later use of IL17Ai post-TNF inhibitor or a more resistant disease phenotype. These results contribute to the body of real-world evidence confirming the role of IL-17i in the management of axSpA and PsA.

## Data Availability

Data are available upon reasonable request. All data relevant to the study are included in the article.
